# Married couples’ dynamics, gender attitudes and contraception use in Savannakhet Province, Lao PDR

**DOI:** 10.1080/16549716.2020.1777713

**Published:** 2020-08-03

**Authors:** Ketmany Chanthakoumane, Charlotte Maguet, Dirk Essink

**Affiliations:** a Lao Tropical and Public Health Institute, Vientiane, Lao PDR; b Vrije Universiteit Amsterdam, Amsterdam, Netherlands; c Athena Institute, Vrije Universiteit Amsterdam, Amsterdam, Netherlands

**Keywords:** LEARN: Sexual Reproductive Health, ANC and Nutrition, Contraceptive methods, family planning, gender inequalities, contraceptive knowledge, decision making

## Abstract

**Background:**

The use of contraception in Lao PDR remains inadequate. In 2017, unmet contraception needs among married women aged 15–49 were 14.3% in Lao PDR overall and 18.6% in the province of Savannakhet. Although the government has a goal to reduce gender inequalities, they still persist in many areas.

**Objective:**

The aim of this research was to understand the extent to which couples’ dynamics and gender attitudes affect contraception use in Savannakhet, Lao PDR.

**Methods:**

To conduct this research, mixed methods were used. Quantitative methods took the form of a survey filled out by 200 married couples in the province of Savannakhet. Afterwards, focus group discussions were carried out to give meaning to the quantitative data and to obtain a deeper understanding of gender roles and contraceptive use.

**Results:**

Findings showed that most couples rely on female-dependent contraceptives and that while women hold most of the family planning responsibility, men’s opinions have more weight on the final decision. Additionally, women’s financial autonomy and spousal communication regarding birth control were associated with contraceptive use within the couple. However, this communication usually began after the birth of the third child. Lastly, the hypothesis that egalitarian gender attitudes were associated with contraceptive use could not be confirmed.

**Conclusion:**

This study clearly demonstrates that contraception use is influenced by couples’ dynamics, more specifically spousal communication, in Lao PDR. The findings have highlighted the need to involve men in all stages of family planning, and to foster both spousal communication and financial autonomy for women. If the findings are implemented, this may foster shared decision making within couples.

## Background

The use of modern methods of contraception among married women aged 15–49 in Lao PDR was 54.1% in 2017, and many contraceptive needs remain unmet [1]. These needs include contraception for both birth-spacing and for limiting births and affect 14.3% of married women aged 15–49 [1]. Unmet needs for contraception have an impact on both maternal and child health. Unwanted births have been linked to higher risks of maternal and child health problems, and thus contribute to maternal and infant mortality [2]. Recently, a rise in contraception use and longer birth-spacing has been associated with an observed decrease in maternal mortality [3]. Family planning is often discussed and researched as a women’s issue. However, studies have demonstrated that contraception use is influenced by inter-couple gender attitudes and dynamics such as decision making and communication.

For example, research investigating couple dynamics in the choice for contraceptives have highlighted power imbalances between partners. Literature from Ethiopia, Ghana, and Kenya has found that husbands have more influence on the decision to use contraception than their wives [4–6]. In addition, in Kenya, communication between partners about desired number of children and family planning use was associated with higher contraceptive use [7]. In Bangladesh high decision-making power of women regarding household issues was associated with contraceptive use [8]. An association between couple’s joint decision making and contraceptive use was observed in Pakistan [9].

In addition, it was found that gender attitudes within couples were also associated with contraception use but results were heterogenous. A 2013 study on associations between perceptions on gender among married couples and contraception use in Tanzania found that more egalitarian gender attitudes among women were associated with higher contraceptive use [10]. The husbands’ views on gender were, however, not significantly associated with their wives’ contraceptive use. The latter was contrary to results from a 2014 study conducted in India. The authors found that married egalitarian gender attitudes and gender sensitive decision making, meaning that the wife takes part in the decision on issues that affect her or her family, among married men were both associated with current contraceptive use [11]. There is no consensus regarding associations between gender attitudes and contraception use in all low- and middle-income countries and research should be performed at country level to account for local culture.

**Figure 1.**
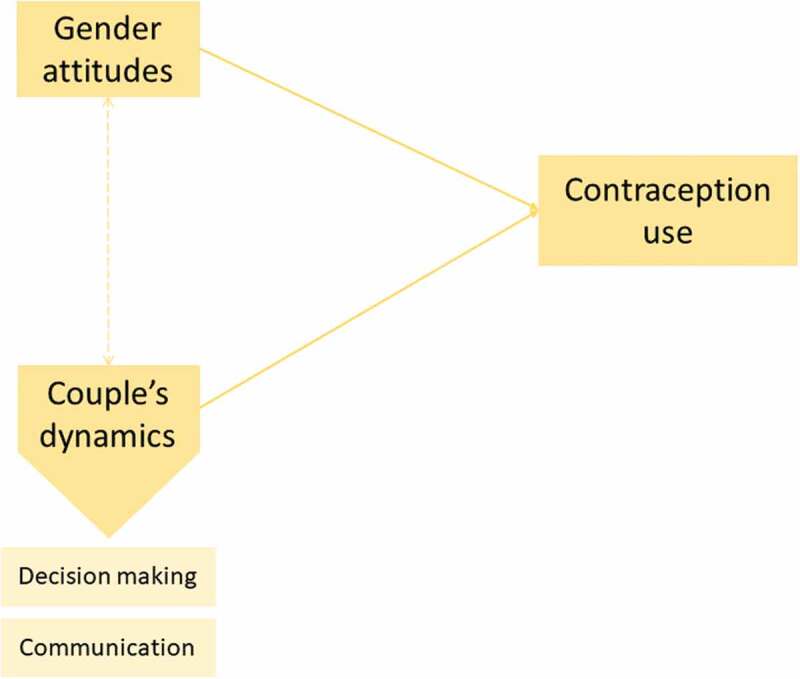
Conceptual model used for the research.

In Lao PDR, a recent study about married couples’ perceptions on women’s autonomy and subjective well-being discussed couples’ dynamics and more specifically decision making. In fact, self-reported autonomy by wives was associated with higher well-being of the women. However, the husbands’ perceptions of their wives’ autonomy showed no association with the well-being of women [12]. Despite this recent insight in decision making in Lao PDR, and although couples’ dynamics, gender attitudes, and contraception use have been studied internationally, no study addressing any of the first two notions in relation to contraception use been made in the country. Thus, the aim of this research is to understand the extent to which couples’ dynamics and gender attitudes affect contraception use in Savannakhet Province, Lao PDR. This was done in two steps. Firstly, contraception use and demographics in the province of Savannakhet were studied. Secondly, the extent to which gender attitudes and couple’s dynamics, namely decision making and spousal communication, determine contraception use was investigated. A model has been developed to investigate the relationship between the concepts previously described (Figure 1). This research could provide new insights on what influences the contraception decision at a couple’s level. Such knowledge on barriers to contraceptive use could help to empower couples, particularly women, to make balanced choices regarding matters of reproduction.

## Methods

### Study design

This research followed a mixed-methods cross-sectional explanatory approach to obtain insights into gender and its influence on the use of contraception, using a survey and focus group discussions (FGDs). Qualitative methods were used to confirm the quantitative data and to get a deeper understanding of gender’s influence on contraception use in Savannakhet Province.

### Study site, population and recruitment

**Figure 2.**
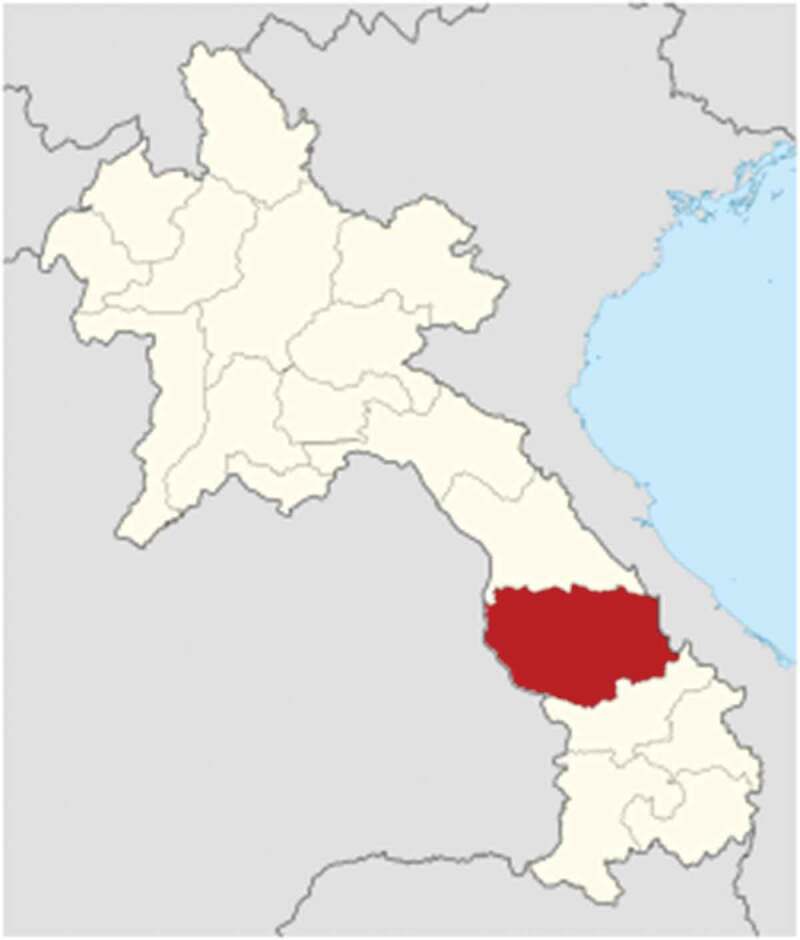
Savannakhet province (in red) in Lao PDR. Source: Wikipedia.

Survey participants were recruited in 2 districts and 10 different villages in Savannakhet province (Figure 2). This province was chosen because unmet contraception needs there (18.6%) are higher than the national average (14.3%) [1]. The two districts were purposefully chosen for their opposite characteristics. Outhoumphone, is an urbanised district, while Seponh is more rural. This allowed for comparison of contraception use in different settings. Ten villages in total, 5 per district, were randomly selected from a list of villages provided by the district health centres. Next, couples were selected randomly from a list of households obtained from the heads of villages. This list contained all eligible couples that met the inclusion criteria, namely opposite-sex married couples with women being of reproductive age, between 15 and 49 years old. Twenty couples were selected per village by the data collection team, which added up to 40 participants per village, and to 400 participants in total. The sample size was calculated using a web-based sample size calculator. The sample size obtained was 384 participants, based on a CI of 95%, a margin error of 5%, a population of 969.697 (Savannakhet province) and a sample proportion likelihood of 50%, and was rounded up to 400. Lastly, to be included in the study, both partners had to agree to participate.

Four FGDs in total and 2 per district, one with men and one with women, were conducted. One village per district was selected randomly from the previously mentioned list to conduct the two FGDs. The study population comprised 29 participants divided in 4 FGDs. The eligibility of participants was similar to that of the survey. However, the participants of the male and female FGDs were not required to form couples. The participants were selected by the head of the village and by Lao Women’s Union based on eligibility and availability of people at the time. Two FGDs were executed in each district to allow for a separation by gender and thus ensure that the participants would be comfortable addressing sexuality and contraception [13]. The first FGD comprised 7 women, the second one 7 men, the third 7 women and the fourth 8 men. Homogeneity was high in the FGDs as participants of each group were from the same village and same sex. The participants were acquainted.

### Data collection tools

#### Questionnaire design

The survey tool combined several questionnaires found in the literature and was adapted to the context of the research. It addressed the following topics: participants’ characteristics, gender role perceptions [14,15], decision making [16,17], spousal communication [17], reproductive health situation [18], and family planning and contraception [19]. Gender role perceptions were assessed with a gender role scale, composed of two sub-scales that are oriented differently [14,15]. In the gender-transcendent scale the respondents were asked whether they agreed or disagreed with general statements oriented towards gender equality such as ‘People should be treated the same regardless of sex’ and ‘Tasks around the house should not be assigned by sex’. The gender-linked scale was composed of statements linking certain behaviours or tasks to a specific gender, for instance: ‘A father’s major responsibility is to provide financially for his children’ and ‘Some types of work are just not appropriate for women’. Lastly, gender role perception was also assessed with questions regarding wife battering. Participants were asked whether wife battering is acceptable in six scenarios. Regarding family planning and contraception, participants were asked whether they have ever used a method of contraception, whether they were using one at the time of the survey, and if so, which one.

The questionnaire was initially created in English and translated to Lao. Eventually, the survey was piloted in Thoulakhom district in Vientiane province and adapted accordingly.

#### Focus group discussion design

The FGDs were designed in a structured manner to validate answers from the questionnaire and try to explain these. It was thus developed parallel to the analysis of the quantitative data. The FGDs addressed the contraception decision within the couple and spousal communication to obtain in-depth information that the questionnaire alone could not provide. Activities were also developed to facilitate the communication around difficult topics. The FGDs were also initially designed in English and translated in Lao.

### Data collection process

Quantitative data was collected in March 2019 and qualitative data in April/May 2019. Two teams of employees of the LaoTPH Institute were assigned to the two different districts and each one comprised two men and two women to ensure that participants would always be interviewed by someone of their gender. The questionnaires were filled out by the interviewers asking the questions in Lao language to the participants and explaining or reformulating questions when needed. Most participants understood Lao language, but when necessary, the interviewers would ask a worker from the health centre to translate. Each participant was interviewed individually, whether at home, at the head of the village’s office or at the temple, to ensure privacy and to prevent a potential influence of the spouse on the respondent’s answers. Two months later a smaller team conducted the FGDs. This team comprised the authors KC and CM who were further assisted by another interviewer from the LaoTPHI. During the FGDs, the facilitator asked the questions in Lao language and the participants conversed together in ethnic languages before translating their ideas to Lao again to be understood by the members of the data collection team.

### Data analysis

All quantitative and qualitative data was translated from Lao to English when necessary and entered into SPSS for analysis. Descriptive statistics were performed to gain insights into perspectives, experiences and behaviours of respondents, such as contraceptive use. From the respondents’ answers, a new variable on contraception use in couples was made. It was coded *yes* if at least one person per couple had answered that they were using a method of contraception at the time of the survey. Then, inferential statistics were done with binary multiple logistic regressions to look for associations. The dependent variable always corresponded to the use of contraceptives within couples, while demographics, decision making, and gender attitudes were used as independent variables in different regression analyses. Five demographic variables were utilised in a first regression analysis, namely age, district, educational attainment, occupation and income. Other demographic variables, such as ethnicity and religion, were excluded as they correlated too much with the district variable. A separate regression analysis was done for decision making and included several independent variables on financial issues, on permission for women to leave the house, and on family planning issues. Concerning spousal communication, another regression analysis included two independent variables: communication on desired number of children and on birth control. Regarding gender attitudes, the consistency of both scales was tested using a Cronbach’s alpha reliability test. Each scale was made into a composite score, and one other variable on wife-battering behaviour was used in a last regression analysis. The scores of each scale were divided in three equal parts and corresponded to low, medium, and high scores respectively. The level of acceptability of wife battering was measured with six scenarios. It was coded *never* for acceptable in no scenarios, *some cases* for one to four out of the six scenarios, and *most cases* for five or all scenarios. Lastly, most variables were stratified per gender during the analysis.

FGDs transcripts were translated from Lao to English, and analysis was done using *Atlas.ti*. Data saturation was achieved after four FGDs. The conceptual model was used for closed coding. Additionally, open coding was performed to obtain new insights into people’s gendered experiences in relation to contraception use.

### Ethical considerations

Ethical approval for the study was obtained from the National Ethics Committee for Health Research of Lao PDR. To ensure that the study was performed in an ethical manner, informed consent forms were obtained from all participants filling out the questionnaires. When participants were unable to read, the interviewers would read the document to them out loud. And participants who could not write were offered the possibility to sign the consent form using a stamp. Oral consent was given by all participants of the four FGDs. Data were anonymized and results do not include any information that may make the data traceable to the participant.

## Results

Most respondents were in the age range 25–44, Buddhist, had achieved primary school, and worked as farmers (Table 1). The urban district Outhoumphone comprised mostly Lao-Tai people (96.9%), while the rural district, Seponh, comprised 62.0% of Mon-Khmer people. Almost 60% of participants were using a contraceptive method with their partner. Most of the contraceptive methods used were female-dependent, the most used being injection (44.4%) and the pill (31.6%).

**Table 1.** Count, frequencies and odds ratio of demographics variables (adjusted for age, district, educational attainment, occupation, and income).

Variables		N (%)	% using contra-ception within couple	Significance in relation to contraception use	p-value	95% CI Lower	95% CI Higher
*Contraceptive use within couple^a^*	No	162 (41.5%)	-	-	-	-	-
	Yes	228 (58.5%)	-	-	-	-	-
	Total	390 (100.0%)					
*Types of contraceptive used*	Injection	52 (44.4%)	-	-	-	-	-
	Contraceptive pill	37 (31.6%)	-	-	-	-	-
	Implant	10 (8.5%)	-	-	-	-	-
	Female sterilisation	6 (5.1%)	-	-	-	-	-
	IUD	6 (5.1%)	-	-	-	-	-
	Condoms	5 (4.3%)	-	-	-	-	-
	Natural methods	4 (3.4%)	-	-	-	-	-
*Demographics^b^*							
*Gender*	Male	200 (50.0%)	-	-	-	-	-
	Female	200 (50.0%)	-	-	-	-	-
	Total	400 (100.0%)					
*Ethnicity*	Lao-Tai	265 (67.1%)	63.6%	-	-	-	-
	Mon-Khmer	130 (32.9%)	46.9%	-	-	-	-
	Total	395 (100%)					
*Religion*	Buddhism	261 (65.7%)	63.2%	-	-	-	-
	Spiritualism^c^ and others	136 (34.3%)	49.3%	-	-	-	-
	Total	397 (100%)					
*District*	Outhoumphone (urban)	200 (50.0%)	63.5%	1(ref)			
	Seponh (rural)	200 (50.0%)	53.5%	1.558	0.131	0.877	2.769
	Total	400 (100%)					
*Age*	19 and under	26 (6.5%)	37.5%	0.455***		0.173	1.192
	20–24	48 (12.0%)	41.7%	0.527***		0.265	1.048
	25–34	148 (37.0%)	53.8%	1 (ref)	0.000		
	35–44	137 (34.3%)	73.1%	2.431***		1.411	4.186
	45 and above	41 (10.3%)	59.0%	1.259***		0.583	2.716
	Total	400 (100%)					
*Educational attainment*	No education	121 (30.3%)	52.1%	0.740	0.257	0.440	1.245
	Primary school and higher	278 (69.7 %)	61.1%	1 (ref)			
	Total	399 (100%)					
*Occupation*	Farmer	355 (89.6%)	57.3%	1(ref)			
	Other	41 (10.4%)	69.2%	1.504	0.319	0.674	3.357
	Total	396 (100%)					
*Income*	Low (<6 M kip)	209 (53.0%)	51.2%	1 (ref)			
	High (>6 M kip)	185 (47.0%)	66.1%	1.761*	0.024	1.079	2.875
	Total	394 (100%)					

*^a^* Contraception use within couple means any partner can be using contraception.

*^b^* Age, district, educational attainment, occupation, and income were all in the same regression analysis. However, ethnicity and religion were not included in the regression analysis since they were too correlated to the district.

*^c^*Spiritualism is an overarching term for the several ethnic religions practiced in Lao.

^*^Significant at p value <0.05. **Significant at p value <0.01. ***Significant at p value <0.001

A binary multiple logistic regression showed that out of age, educational attainment, district, ethnicity, income, and occupation, both age and income were significantly associated with contraception used by the couple (Table 1). Regarding age, people in the 35–44 age range had 2.43 (95% CI 1.41 to 4.19) times greater odds of using contraception than those in the 25–34 age range. On the other hand, people who were 19 and under had 0.46 (95%CI 0.17–1.19) odds of using contraception compared to people in the 25–34 age range. In addition, descriptive statistics showed that people in the age range 35–44 were the ones using contraception the most (Table1). Concerning income, the odds of using contraception among the couple were 1.76 (95% CI 1.08–2.88) greater for the high-income group compared to low income one (Table1).

### Gender attitudes

On the gender-transcendent scale, 73.8% of people scored high, which corresponds to having egalitarian views between genders, while 22.9% and 3.3% scored medium and low respectively. However, on the gender-linked scale, 75.3% scored low, which corresponds to having inegalitarian views and to assigning specific roles to each gender (Table 2). Lastly, 23.4% of participants scored medium and 1.3% high. No major difference was observed when stratified per gender for the gender-transcendent and the gender-linked scale (Table 2). Most respondents (60.6%) considered wife battering acceptable in one to four of the six scenarios suggested in the questionnaires, while only a small proportion (12.2%) never considered it acceptable. A similar proportion of men and women never considered wife battering acceptable, but more than twice as many women (41%) as men (15%) found it acceptable in most cases (Table 2).

**Table 2.** Count, frequencies and odds ratio of gender attitudes and decision making.

Variables			N (%)	% using contra-ception within couple	Significance in relation to contra-ception use	p-value	95% CI Lower	95% CI Higher
*Gender attitudes^a^*								
*Gender transcendent scale^b^* *(gender equality-oriented scale)*	Women	Low	9 (4.6%)	44.4%	1.420		0.188	10.721
		Medium	36 (18.6%)	68.6%	2.622		0.905	7.596
		High	149 (76.8%)	56.6%	1(ref)	0.206		
	Men	Low	4 (2.0%)	75.0%	582,612,039.8		0.000	-
		Medium	54 (27.1%)	54.7%	0.813		0.372	1.776
		High	141 (70.9%)	59.1%	1(ref)	0.874		
	**Total**	Low	13 (3.3%)	53.8%	1.576		0.352	7.067
		Medium	90 (22.9%)	60.2%	1.107		0.618	1.984
		High	290 (73.8%)	57.8%	1(ref)	0.816		
*Gender-linked scale^b^ (oriented towards gender-specific roles)*	Women	Low	140 (74.9%)	60.1%	1(ref)	0.954		
		Medium	44 (23.5%)	57.1%	0.873		0.364	2.095
		High	3 (1.6%)	33.3%	0.965		0.029	32.528
	Men	Low	146 (75.6%)	58.0%	1(ref)	0.124		
		Medium	45 (23.3%)	65.1%	2.526		1.038	6.149
		High	2 (1.0%)	0.0%	0.000		0.000	-
	**Total**	Low	286 (75.3%)	59.1%	1(ref)	0.133		
		Medium	89 (23.4%)	61.2%	1.410		0.780	2.549
		High	5 (1.3%)	20.0%	0.149		0.11	2.006
*Wife battering considered acceptable*	Women	Never	18 (10.2%)	70.6%	1.180		0.292	4.767
		In some cases,	86 (48.9%)	59.8%	1(ref)	0.941		
		In most cases	72 (40.9%)	51.4%	0.926		0.433	1.979
	Men	Never	27 (13.7%)	57.7%	0.798		0.305	2.086
		In some cases	140 (71.1%)	61.6%	1(ref)	0.130		
		In most cases	30 (15.2%)	46.4%	0.378		0.147	0.974
	**Total**	Never	45 (12.2%)	62.8%	0.926		0.443	1.937
		In some cases,	226 (60.6%)	60.9%	1(ref)	0.450		
		In most cases	102 (27.3%)	50.0%	0.704		0.409	1.213
*Decision making*^c^								
*On financial issues*	Women	Woman involved	153 (76.9%)	64.2%	1 (ref)			
		Woman not involved	46 (23.1%)	39.1%	0.410*	0.046	0.171	0.983
	Men	Woman involved	137 (68.5%)	60.0%	1 (ref)			
		Woman not involved	63 (31.5%)	55.0%	0.975	0.954	0.408	2.326
	Total	Woman involved	290 (72.7%)	62.2%	1 (ref)			
		Woman not involved	109 (27.3%)	48.1%	0.638	0.130	0.356	1.141
*Women’s freedom to go outside*	Women	No freedom	159 (83.7%)	56.8%	1 (ref)	0.977		
		Low freedom	18 (9.5%)	72.2%	0.960		0.268	3.434
		High freedom	13 (6.8%)	61.5%	0.872		0.245	3.102
	Men	No freedom	159 (79.5%)	55.8%	1 (ref)	0.506		
		Low freedom	33 (16.5%)	66.7%	0.587		0.194	1.773
		High freedom	8 (4.0%)	75.0%	0.386		0.031	4.840
	Total	No freedom	318 (81.5%)	56.3%	1 (ref)	0.731		
		Low freedom	51 (13.1%)	68.6%	0.755		0.336	1.694
		High freedom	21 (5.4%)	66.7%	0.770		0.265	2.239
*On the number of children to have*	Women	Woman involved	169 (84.5%)	58.2%	1 (ref)			
		Woman not involved	31 (15.5%)	60.0%	1.218	0.701	0.445	3.334
	Men	Woman involved	171 (88.1%)	56.9%	1 (ref)			
		Woman not involved	23 (11.9%)	63.6%	1.865	0.428	0.400	8.692
	Total	Woman involved	340 (86.3%)	57.5%	1 (ref)			
		Woman not involved	54 (13.7%)	61.5%	1.380	0.438	0.611	3.116
*On contra-ception use*	Women	Woman only	38 (23.0%)	76.3%	1.504	0.403	0.578	3.911
		Couple or other	127 (77.0%)	61.8%	1 (ref)			
	Men	Woman only	30 (23.4%)	58.6%	0.816	0.668	0.321	2.072
		Couple or other	98 (76.6%)	61.5%	1 (ref)			
	Total	Woman only	68 (23.2%)	68.7%	1.052	0.876	0.556	1.992
		Couple or other	225 (76.8%)	61.6%	1 (ref)			
*Communication^d^*								
*On desired number of children*	Women	Often discussed	127 (63.8%)	62.6%	1 (ref)	0.007		
		Not very often discussed	43 (21.6%)	37.2%	0.511**		0.180	1.450
		Never discussed	29 (14.6%)	71.4%	3.937**		1.213	12.779
	Men	Often discussed	85 (42.5%)	53.7%	1 (ref)	0.189		
		Not very often discussed	98 (49.0%)	65.6%	1.491	0.337	0.660	3.371
		Never discussed	17 (8.5%)	41.2%	0.495	0.283	0.137	1.785
	Total	Often discussed	212 (53.1%)	59.0%	1 (ref)	0.446		
		Not very often discussed	141 (35.3%)	56.8%	1.110	0.743	0.595	2.068
		Never discussed	46 (11.5%)	60.0%	1.678	0.207	0.751	3.751
*On birth control*	Women	Often discussed	109 (55.9%)	71.4%	1 (ref)	0.000		
		Not very often discussed	29 (14.9%)	62.1%	0.911***		0.291	2.852
		Never discussed	57 (29.2%)	33.9%	0.167***		0.065	0.433
	Men	Often discussed	44 (22.4%)	64.3%	1 (ref)	0.004		
		Not very often discussed	103 (52.6%)	68.6%	1.055**		0.397	2.806
		Never discussed	49 (25.0%)	31.9%	0.280**		0.103	0.763
	Total	Often discussed	153 (39.1%)	69.4%	1 (ref)	0.000		
		Not very often discussed	132 (33.8%)	67.2%	0.837***		0.424	1.649
		Never discussed	106 (27.1%)	33.0%	0.210***		0.110	0.401

*^a^*Gender-linked scale, gender-transcendent scale, and wife battering variable were in the same regression analysis (adjusted for age and income).

*^b^* Cronbach’s alpha of 0.75 and 0.79 for the gender transcendent scale (5 questions) and the gender-linked scale (8 questions) respectively, indicating the adequate reliability of both scales.

*^c^* Financial decision making, women’s freedom to go outside, decision making on number of children, and decision making on contraception use were in the same regression analysis (adjusted for age and income).

*^d^* Both variables on communication were in the same regression analysis (adjusted for age and income)

^*^Significant at p value <0.05. **Significant at p value <0.01. ***Significant at p value <0.001.

A binary multiple logistic regression including both sub-scales on gender perceptions and the wife battering variable as independent variables, showed no significant association between gender attitudes and contraception use by couples.

Qualitative data has highlighted that contraception use could still be tied to some gender norms. This was illustrated by the following quote from one of the men of the FGDs:

Contraception is women’s duty because we, men, cannot get pregnant. It is their responsibility to use contraception

This perspective was shared by men in both FGDs, implying that women hold this responsibility because of their gender, although this does not make them decision makers (see next section). Such responsibility confirms the quantitative results showing that most contraception methods used by couples were female-dependent. Men provided additional arguments why they did not use male-based contraceptives. Most prominently it emerged that they do not like condoms, associate them with extra-marital relationships, and fear side effects of male sterilisation. To illustrate: *‘Condoms are not comfortable to use, and sex doesn’t feel the same’. ‘If I were to get sterilised, I feel like I would lose my energy and couldn’t work hard anymore’. ‘If I use condoms, my wife will think that I am cheating on her.’*

### Decision making

In relation to decision making in the household both men and women answered at similar rates that women have to ask their husbands for permission to go out in 87% to 95% of cases. Regarding decision making on financial issues, women believe they are more often involved in the decision making (76.9%) than men believe that women are (68.5%) (Table 2). Lastly, 61.5% of participants stated that women are involved in decisions related to family size in their couple, and 23.2% said that the contraception decision was taken by the woman only.

Binary multiple logistic regression analyses have shown that none of the decision making variables are associated with contraception use in the couple, except financial autonomy reported by women (Table 2). A regression analysis among women only has indicated that women who report not being involved in the household decision making regarding finance had 0.41 (95% CI 0.17–0.98) odds of using contraception in their couple compared to women who reported being involved in such decision making.

Qualitative analysis gave additional insight on the decision-making process. The FGD in general confirmed that women are, to a certain extent, involved in the contraception decision. Contraception use has been described by men as women’s duty. Women described often taking the responsibility to initiate decision making to use contraception, for example when they thought another child would create financial problems. Another illustration of the responsibility that women hold regarding family planning can be found in the following quote by an urban woman: *‘My husband refuses to use condoms and he says that it would be fine if I got pregnant’.*

Despite this responsibility, most women indicated that they need to consult their husbands before using contraception, as they do not dare to decide by themselves. Most women of the rural district further indicated that they have to accept their husband’s decision even if it means not using any contraception. Urban women described that if their husband refused that they should use contraceptives, the women would negotiate with them or use a contraception secretly. The men of the urban FGD acknowledged that they cannot force their wives.

### Spousal communication

More than half of the respondents (53%) stated they often discuss the number of children to have with their partner (Table 2). Contraception use was discussed less often, 39% of participants answered that they often discuss birth control with their partners. When stratified per gender, major differences were observed between men’s and women’s answers, 63.8% of women reported often discussing about the desired number of children against 42.5% of men. Regarding birth control, 55.9% of women stating often discussing the issue while only 22.4% of men reported the same frequency. Binary multiple logistic regression adjusted for age and income has shown that participants who never discussed birth control and those who did not discuss it very often had respectively 0.21 (95% CI 0.11 to 0.40) and 0.84 (95% CI 0.42 to 1.65) odds of using contraception compared to those who often discussed it (Table 2). Gender stratification has revealed significant associations between communication on birth control and contraception use among both men and women **(see** Table 2 **for OR)**. Regarding discussion of the desired number of children, women who reported never discussing it and those who reported it not discussing it very often had respectively 3.94 (95% CI 1.21 to 12.8) and 0.51 (95% CI 0.18 to 1.45) odds of using contraception in their couple compared to women who never discussed family size with their partner.

Qualitative data on spousal communication revealed that the question of contraception was usually not addressed before marriage and that couples do not use any method during this time. In addition, it was explained by participants of both genders that the desired number of children is rarely discussed in a relationship before the third child is born. Men added that they generally prefer to have more children as they can help with work in the family.

## Discussion

This study set out to understand the relationships between gender attitudes, couples’ dynamics and contraceptive use. Contraception use is not evident for many people in Savannakhet Province, Lao PDR. Less than 60% of people were using contraception, which is similar to national statistics [1]. Both age and income were associated with contraception use. The finding that people in the 35–44 age range use contraception the most are in line with the results of the 2017 demographic survey stating that people aged 35–39 are the main users [1]. Contrary to other studies, we could not confirm that rural women, when adjusted for income, use contraceptives less than urban women [20].

Our hypothesis that more egalitarian gender attitudes were associated with higher contraceptive use could not be confirmed. Our findings do confirm that respondents have inegalitarian gender attitudes in practice and tend to assign specific roles to each gender, despite having egalitarian views in theory. More research in Lao PDR is recommended to investigate gender inequalities at structural and individual levels and their effects on contraceptive use. In addition, and similarly to other studies in Lao PDR, wife battering was considered acceptable in some or even most of the cases by the majority of respondents [21,22].

The result that spousal communication was also associated with contraception use within couples is in line with other studies [7,23]. Interestingly, discrepancies were found between men and women, indicating a possible difference in perception of frequency of discussions or the presence of a desirability bias. Further research is recommended to investigate these discrepancies between genders, and to explore possible interventions to foster spousal communication. We report mixed findings in decision making within couples on contraceptive use. The survey revealed that it is often the couple, or the women who makes the decision. In the FGDs it became apparent that men often make the final decision, but that it is the responsibility of women to initiate the discussion. Especially in rural areas, women appear to accept their husbands’ decisions, even if it means not using contraception. This is also exemplified by the fact that male-based contraceptives are hardly utilised among couples; condom use is rarely accepted by men within marriage; male-sterilisation is not reported in our study population nor in Lao PDR [1], while female sterilisation accounts for 5% of the contraceptive methods used, even though this is less cost-effective and presents higher rates of complications [24,25]. This is similar to other countries in the world, where family planning responsibility often lies with women and many programmes promoting contraceptive use focus on women [26,27]. We hypothesise that, despite mixed findings in the quantitative results, in Lao PDR women are largely responsible for the planning regarding contraceptive use and its actual use, but men are equally or more dominant in relation to deciding on the use of contraception. Women who indicated having high financial decision-making power, or autonomy, seemed to be the exception. They were more likely to use contraceptives, regardless of the husband’s perception of his wife’s financial autonomy.

Based on the findings regarding decision making and the effect of spousal-communication, the researchers emphasize the need for including men in programmes concerning decision making on contraceptives. Several studies have shown the positive impact of involving men in family planning [28–32]. Involving men in family planning can be done using a gender-accommodating or gender-transformative approach depending on the desired outcome [33]. However, women should not be overlooked by programmes since they too are agents of patriarchy and support gender norms [10]. Additionally, the results show the importance of programmes that promote women’s financial autonomy, in a context where reaching financial independence remains difficult for women for structural and cultural reasons [34].

### Strengths and limitations

A strength of this research is the inclusion of couples’ dynamics to study possible influences on contraception use. In fact, studies on contraception use often focus on women only, excluding the influence of the partner and of the relationship [35,36]. Another important issue brought to light by this study is the underlying gender inequalities that rule people’s lives. Despite this, gender remains understudied and the terms ‘gender’ and ‘sex’ are even regularly confused in research. It is important to distinguish between the two to increase rigour in research [37,38]. In addition, the sample population is reasonably representative of the population of the province of Savannakhet since the research included enough participants and most questions had a high participation rate. Moreover, adequate Cronbach’s alpha coefficients ensured that the gender perception scales were reliable (Table 2). Our mixed methods approach allowed us to unravel quantitative findings. For example, the FGDs changed our perspective on what ‘shared’ decision making on contraceptives meant.

This research is cross-sectional which prevents inferring any causal links. Therefore, only associations can be confirmed. In addition, results being translated from ethnic languages to Lao and then from Lao to English reduced the quality and quantity of results. Another limitation of this research was that, although the participation rate was generally high, a mistake in questionnaire design lowered the rate of the question on contraception decision to 75%.

## Conclusion

This study demonstrates that contraception use is influenced by couples’ dynamics in Lao PDR. Male-based contraceptives are hardly used, women are making plans for contraceptives, yet males are quite dominant in deciding on the use of contraceptives. In addition, spousal communication on birth control and women’s financial autonomy are associated with contraception use. This research brings new insights that can help in the journey to shared decision making on contraception within couples. Possibilities to apply the findings of this research to the real world include involving men in family planning, fostering spousal communication, and promoting financial autonomy among women. Lastly, more research should be carried out on interventions to improve spousal communication and on how gender inequalities at structural and individual level affect contraception use.

## Paper context

Gender inequalities persist in Lao PDR and unmet contraception needs remain too high. This research aims to understand the relationships between gender attitudes, couples’ dynamics and contraceptive use. It shows that decision making on contraception is unbalanced in couples, that financial independence for women and spousal communication on birth control are associated to contraception use. Thus, programmes should thrive to involve men in family planning, foster spousal communication, and promote financial autonomy for women.
